# Draft Genome Sequence of a Phosphate-Accumulating *Bacillus* sp., WBUNB004

**DOI:** 10.1128/genomeA.00251-12

**Published:** 2013-02-21

**Authors:** Shreya DebRoy, Pallavi Mukherjee, Sujata Roy, Ashoke Ranjan Thakur, Shaon RayChaudhuri

**Affiliations:** a West Bengal University of Technology, BF-142, Sector 1, Salt Lake, Kolkata, West Bengal, India; b Rajalakshmi Engineering College, Thandalam, Chennai, India; c Techno India University, EM4/1, Sector V, Salt Lake, Kolkata, West Bengal, India

## Abstract

The draft genome sequence of a nitrate- and phosphate-removing, Gram-positive *Bacillus* sp. with optimum growth at 37°C and pH 7 in nitrate broth (HiMedia M439) isolated from rhizosphere of a water lily, with a genome size of 5,465,157 bp and a G+C content of 35.0%, is reported here.

## GENOME ANNOUNCEMENT

Approximately 76% of the world’s population lives in developing countries where more nitrogen fertilizer is applied, resulting in increased nitrate pollution of groundwater. Investigations in developing countries attribute this pollution to inefficiently managed nitrogen fertilizer application (1). A high concentration of nitrate in drinking water is a threat, especially to infants, causing methemoglobinemia. The carcinogenic effect of nitrate has also been reported; concentrations higher than 10 ppm in drinking water might also cause stomach cancer in infants (2).

Phosphorus, though recognized as one of the major nutrients for the survival of living organisms, causes environmental pollution at high concentrations, contributing to the eutrophication of water bodies. The major sources of phosphates in the environment are detergents, fertilizers, and sewage (3). High levels of phosphate in water can cause digestive problems, whereas levels of >1 ppm might interfere with coagulation in water treatment plants (4).

In a search to find a microbe that removes nitrate and phosphate from wastewater, several environmental sites (5) were screened. A *Bacillus* sp. from the rhizosphere of a water lily might survive at 2,000 ppm of nitrate, with a generation time of 21.4 min in nitrate broth (HiMedia M439). It removed 54.6% nitrate within 16 h. Approximately 83% of phosphate was removed as compared to *Acinetobacter baumannii* MTCC 1425 (~32%) under similar conditions (5). The strain is available at the Microbial Culture Collection at the National Centre for Cell Sciences in Pune, India.

The whole-genome sequencing was performed on an Ion Torrent personal genome machine (PGM) instrument on a 316 chip. The reads were assembled using MIRA Assembler v3.4.0. The total number of assembled reads was 1,602,103, with a coverage of 40×. There were 127 contigs. A total of 265 Mb of data was sequenced, with 157 Mb having a quality value >20. All contigs generated by MIRA assembly were submitted to the Rapid Annotations using Subsystem Technology RAST (6) server. The Mauve-based analysis of the contigs revealed extensive rearrangement of the genome compared to other members of the genus *Bacillus*.

The genes for nitrite reductase [NAD(P)H] large and small subunits, nitrate/nitrite transporter, and respiratory nitrate reductase-alpha, -beta, -gamma, and -delta chain, as well as bacteriophage-related protein and phage replication protein, are all carried by accession no. ANGA01000011; the gene for nitrate/nitrite sensor protein was carried by accession no. ANGA01000034; genes for nitroreductase family proteins were carried by accession no. ANGA01000033; and the gene for nitrate/nitrite response regulator protein was carried by accession no. ANGA01000054. The gene responsible for phosphate metabolism, namely, inorganic pyrophosphatase (*ppaX*), was carried by accession no. ANGA01000047, and the genes for polyphosphate kinase (*ppk*), exopolyphosphatase (*ppx*), phosphotransferase system phosphocarrier protein (HPr gene), and phosphate transport system regulatory protein and phosphate transport system permease protein (*pstA*, *pstC*), as well as alkaline phosphatase, were carried by accession no. ANGA01000017. Genes for ATP synthase A, B, and C chains were carried by accession no. ANGA01000040, while the gene for urease domain protein was carried by accession no. ANGA01000009. Walker motifs have been detected in the above two contigs (ANGA01000040, ANGA01000009), emphasizing a role in phosphate metabolism.

### Nucleotide sequence accession numbers.

This Whole Genome Shotgun project has been deposited at DDBJ/EMBL/GenBank under the accession no. ANGA00000000. The version described in this paper is the first version, ANGA01000000.
